# Estimation of genetic parameters for growth and carcass traits in turbot (*Scophthalmus maximus*)

**DOI:** 10.5194/aab-62-265-2019

**Published:** 2019-05-06

**Authors:** Kristina Schlicht, Nina Krattenmacher, Vincent Lugert, Carsten Schulz, Georg Thaller, Jens Tetens

**Affiliations:** 1 Institute of Animal Breeding and Husbandry, Christian Albrechts University of Kiel, Hermann-Rodewald-Straße 6, 24118 Kiel, Germany; 2 College of the Marshall Islands, P.O. Box 1258, 96960 Majuro, Republic of the Marshall Islands; 3 GMA – Gesellschaft für Marine Aquakultur mbH, Hafentörn 3, 25761 Büsum, Germany; 4 Department of Animal Sciences, Functional Breeding Group, Georg August University Göttingen, Burckhardtweg 2, 37077 Göttingen, Germany; 5 Center for Integrated Breeding Research, Georg August University, 37075 Göttingen, Germany; 6 Department of Internal Medicine 1, Kiel University Hospital, Kiel University, Arnold-Heller-Str. 3, 24105 Kiel, Germany

## Abstract

Information on phenotypic and genetic (co)variance for production traits in turbot is required to improve breeding programs. So far, information on morphometric growth traits is sparse and completely lacking on quality carcass traits like fillet weight or fillet yield for turbot. As part of a long-term study we explored the phenotypic and genetic (co)variance of 16 biometrical and carcass traits of three different European turbot strains. Fish were reared under commercial grow-out conditions, including size grading. We used molecular relatedness (MR) methods based on genotyping with 96 microsatellite markers and animal models. We included an adapted condition factor for Pleuronectiformes (FCI${}_{\mathrm{PLN}}$) and average daily weight gain (ADG) between the ages of 300 and 500 d post-hatch (dph) for their potential correlation with body weight at harvest. Heritability estimates for all traits were low to medium (0.04–0.29) when strains were jointly analyzed. Separate analysis of strains yielded higher heritability estimates (0.12–0.43). Genetic correlations between weight-related traits were highly positive (0.70–0.99), while runs with yield and ratio traits often resulted in unreliable estimates of genetic correlation due to high standard errors. Body weight ($h^{2}=0.19$), fillet yield ($h^{2}=0.1$5), and dressing percentage ($h^{2}=0.17$) are particularly promising selection traits for turbot breeding.

## 1 Introduction

Aquaculture production of turbot (*Scophthalmus maximus*) has been steadily increasing over the last years, especially in the PR China (64 000 t in 2013), which is the biggest producer worldwide (FAO, 2012). In Europe, turbot is mainly valued as a high-priced food fish on the upscale gourmet market, with the vast majority of production taking place in Spain and Portugal. Smaller aquaculture facilities for turbot are located in France, Norway, Denmark, and the Netherlands. Turbot is primarily sold whole, with a smaller portion of aquaculture production being sold either gutted or filleted (Turbot Species Leaflet – European Commission, 2016). Regardless of these different marketing strategies, the improvement and harmonization of growth traits remains one of the biggest and economically most relevant challenges of turbot breeding programs. Despite a recent rise in the development of genetic and genomic resources for turbot, reliable estimates of genetic parameters are still needed to improve existing breeding programs. Studies on heritability and genetic correlations in turbot have mostly been focused exclusively on body weight as the trait of the highest economic value (e.g., Gjerde et al., 1997; Guan et al., 2015) and have revealed moderate levels of genetic variation and therefore potential for selection. However, even when fish are marketed whole, traits like fillet weight, fillet yield, and dressing percentage are of great importance with regard to customer preferences and financial profits (Rutten et al., 2005) and have therefore been incorporated as additional selection criteria in many of the established breeding programs for other important aquaculture species (Neira et al., 2004). Additionally, carcass traits like the hepatosomatic index (HSI) and morphometric traits like length, width, or Fulton's condition index (FCI) can be used as readily obtainable indicators for health and fitness conditions. Possible interactions on a genetic level between traits of interest have to be quantified to avoid unfavorable correlated selection response. As far as we know, this is the first time that a quantitative genetic analysis of carcass traits in turbot has been done. As part of a long-term study we explored the genetic and phenotypic (co)variance of 16 biometrical and carcass traits of different turbot strains under commercial grow-out conditions using molecular relatedness (MR) and restricted maximum likelihood (REML) methods. In addition to standard traits like weight and length, we included FCI and average daily weight gain (ADG) between the ages of 300 and 500 d post-hatch (dph) to explore their potential correlation with body weight at harvest. Fulton proposed his condition index to measure fish health in 1904, assuming that the standard weight of the fish is proportional to the cube of its length. Since this assumption is based on the morphometrical traits of roundfish, we additionally included an adapted condition factor for Pleuronectiformes (FCI${}_{\mathrm{PLN}}$) in our analysis. The adapted condition factor relates the weight of the fish to the volume of a hypothetical cube, with its edges defined as $\sqrt{\mathrm{length}\times \mathrm{width}}$, and has been demonstrated by Arfsten et al. (2010) to be much better suited for use in flatfish than the original one.

## 2 Materials and methods

### 2.1 Ethical statement

All experimental animal procedures were carried out following the German Federal Regulations for Animal Protection (§10 Tierschutzgesetzes (TierSchG); §5 Tierschutzversuchstierverordnung (TierSchVersV)).

### 2.2 Experimental design

Nearly 8000 turbot of four different European strains were raised from juveniles to approximately market age in two experiments to collect individual weight and morphometric growth data. During the first trial in 2010–2011 fish originating from Norway (strain A, N=3423) and Iceland (strain B, N=2563) were reared as part of a larger project on growth modeling (Oesau et al., 2013). Each fish was tagged intra-abdominally with passive integrated transponder (PIT) tags (Hallprint, PTY Ltd., Hindmarsh Valley, Australia). Wet body weight (BW, rounded to the nearest gram (g)) and a digital photograph were recorded for each fish individually using a semiautomatic camera setup (Fishback et al., 2002). The experiment lasted for 15 months and measurements were taken in 42 d intervals. Standard length (L), width (W), and area (A) were determined in centimeters from the photographs manually with the ImageJ software (Schneider et al., 2012). Length was defined as length from tail fin to snout, W was measured at the widest point of the fish excluding the fins, and A was defined as the entire body surface area of the fish excluding the fins. Measurement protocols were established in a previous experiment and are described in detail in Oesau et al. (2013). Starting in 2013, animals from France (strain C, N=940) and Norway (strain D, N=1026) were subsequently raised and measured in 30 d intervals for a duration of 18 months, utilizing the same procedures. Both experiments took place in two identical recirculating aquaculture systems (RASs) containing 10 round tanks with a total water volume of 40 m${}^{3}$ each at the Gesellschaft für marine Aquakultur mbH (GMA) in Büsum, Germany. Water temperature was kept constant at 17 ${}^{\circ }$C (±1). Fish were fed a commercial diet (Aller Aqua, Denmark). Further details on feeding and water parameters are described by Lugert (2015) and Oesau (2012). For management reasons fish were graded based on body weight between three and six times during the experiment. This is common practice under commercial grow-out conditions and serves to establish tanks with same-size cohorts to minimize social hierarchies and facilitate feeding management. Each animal was assigned a new number reflecting their contemporary group (CG) after each grading event. CG numbers were unique between the two experiments. CG thus reflects common environment factors such as year, size cohort, and stocking density and has been included as an adjustment in modeling. Gonadal sex and carcass traits were recorded postmortem on an individual basis. After a fasting period of 5 d fish were killed adhering to animal welfare standards using a mixture of ice water and ${\mathrm{CO}}_{2}$, then carved and filleted manually. Since this process was time-consuming and took place on several consecutive days, the age at slaughter differs within strains. Age at slaughter also differs between strains due to nonsynchronized European production cycles, which resulted in different delivery dates and ages of fry. All further analyses have therefore been adjusted to include age at slaughter as a fixed effect. Four filets were cut from each fish, two each on the dorsal and ventral sides. A total of 26 different persons were involved with filleting, and preliminary tests showed large inter-individual variation in fillet yields due to personal experience levels and susceptibility to fatigue. The filleting person (FP) has therefore been recorded for each fish and included as a fixed effect in the models. Individual weights have been collected for fillet without skin (FW), head (HE), liver (LI), gut and stomach (GU), and the rest of the carcass (CA) including bones, skin, and gonads. Total weight of intestines (IW), gutted weight (GW), hepatosomatic index (HSI), fillet yield (FY%), adapted condition index for Pleuronectiformes (FCI${}_{\mathrm{PLN}}$), and dressing percentage (DP%) have been calculated according to the following formulas.

1weight  of   intestines:IW=GU+LI2gutted  weight:GW=BW-IW3hepatosomatic  index:HSI=LI⋅100BW4fillet  yield:FY%=FW⋅100BW5dressing  percentage:DP%=GW⋅100BW6Fulton's  condition  index:FCI=BW⋅100L37adapted  condition  index:FCIPLN=BW⋅100L⋅W328average  daily  gain:ADG*=BW2-BW1dph2-dph1

BW${}_{1}$ is body weight at previous measurement, BW${}_{2}$ is current body weight, dph${}_{1}$ is age in days post-hatching at previous measurement, and dph${}_{2}$ is age at current measurement.

Combining all records from both parts of the experiment resulted in a phenotypic dataset of 7952 turbot, with the larger part originating from the 2010–2011 time period (strain A: N=3423, strain B: N=2593). Since strains A and B were originally part of a larger project with a different research objective the availability of fish for genotyping differed between experiments. In this study genotyping efforts thus had to be focused on animals of strains C (N=940) and D (N=1026). Animals were removed from the dataset if the number of growth recordings per fish was fewer than three (N=69), if gender could not be reliably determined (N=146), or if the animal showed obvious signs of illness based on visual examination during slaughter (N=79); 534 fish of strains A and B had been randomly selected in 2010, resulting in a total number of 2500 genotyped animals. A further 344 individuals had to be excluded later due to poor genotyping quality, leading to a total dataset of 2156 turbot with ages at slaughter ranging from 496 to 758 dph (Table 1). For strain-wise analysis, we excluded stain B due to an overall low contribution to the dataset and combined strains A and D since they originated from the same breeding facility in Norway.

**Table 1:** Numbers, mean weights, and ages of animals at the start of the growth experiment for the complete dataset and for separate strains. N: number of animals; dph: age in days post-hatch; standard deviations are given in brackets.

	Complete	Strain A	Strain B	Strain C	Strain D
	dataset	Norway	Iceland	France	Norway
N	2156	261	250	783	862
Mean body	48.9 (27.4)	108.1 (32.3)	57.1 (21.4)	36.8 (7.9)	39.5 (8.6)
weight (g)					
Mean age	253.6 (46.9)	333.6 (20.4)	256.6 (22.3)	199.3 (4.1)	277.6 (6.2)
(dph)					

### 2.3 Markers and genotyping

Lysates with a high concentration of genomic DNA were produced from fin samples according to a modified protocol by Miller et al. (1988). Fish were genotyped using a panel of 94 polymorphic microsatellite markers (Table S1 in Supplement) that were tested in a small subset of fish and chosen based on their high polymorphism information content. Markers were selected to be evenly distributed across the genome (Bouza et al., 2012), covering 22 of 24 linkage groups with an average inter-marker distance of 10.5 cm. Primer pairs were designed with the Primer 3 software (Untergasser et al., 2012). Primer sequences and marker information are presented in Table S1. Multiplex PCR amplifications were performed using fluorescence labeled primer pairs, the Qiagen Multiplex PCR kit (Qiagen GmbH, Hilden, Germany), and an MJ Research PTC-200 thermal cycler (Global Medical Instrumentation Inc., Minnesota, USA) under the following cycling protocol: initial denaturation at 95 ${}^{\circ }$C for 15 min, 40 cycles each of 94 ${}^{\circ }$C for 30 s, 56–64 ${}^{\circ }$C for 1 h 30 min (depending of the optimal annealing temperature for each multiplex set), 72 ${}^{\circ }$C for 1 h 30 min, and final extension at 72 ${}^{\circ }$C for 20 min. Capillary electrophoresis and fragment length analysis were carried out with an ABI 3130xl Genetic Analyzer and the Genemapper 4.0 software (Thermo Fisher Scientific Inc., USA).

### 2.4 Molecular relatedness (MR)

All data handling and statistical computing were done in R (R Development Core Team, 2011) unless otherwise specified.

As neither pedigree information nor DNA samples of possible parents were available for strains C and D, we estimated molecular relatedness for the total dataset based on co-ancestry using the similarity index described by Eding et al. (2001):

9 $$S_{xy,l}=\frac{1}{4}\left[I_{11}+I_{12}+I_{21}+I_{22}\right],$$

where $S_{xy,l}$ is the similarity index for individuals x and y at locus l, and $I_{ij}$ is an indicator variable that is 1 when allele i at locus l in the first individual equals allele j at the same locus in the second individual; otherwise, it is 0. The values were averaged across loci and according to Hayes and Goddard (2008), and each element of the resulting matrix was transformed as

10 $$S_{xy}=\frac{S_{xy}\;\text{min}}{1\;\text{min}},$$

where min is the minimum similarity in the matrix. This is an estimate of co-ancestry, and molecular relatedness $r_{xy}$ was calculated as $2S_{xy}$ (Lynch, 1988; Blonk et al., 2010). The resulting co-ancestry matrix ($r_{xy}$) was then used to calculate genetic parameters instead of conventional pedigree (PED).

### 2.5 Genetic analysis

For the estimation of heritability we used a univariate additive animal model:

yijklm=μ+STi+CGj+Sk+∑n=05bnxijklmndph11+animall+eijklm,

where $y_{ijklm}$ is the mth observation of BW, L, A, or W, μ is the general mean, ST${}_{i}$ is the fixed effect of strain i (i=1 to 4), CG${}_{j}$ is the fixed effect of contemporary housing group j (j=1 to 64), $S_{k}$ is the fixed effect of gonadal sex k (k=1 (male) or 2 (female)), $b_{n}$(dph) is the fixed regression coefficients on days post-hatch (dph) with $x_{ijklm0}(\mathrm{dph})=$ 1, $x_{ijklm1}(\mathrm{dph})=$ dph, $x_{ijklm2}(\mathrm{dph})=$ $1/2(3\;{\mathrm{dph}}^{2}-1)$, $x_{ijklm3}(\mathrm{dph})=$ $1/2(5\;{\mathrm{dph}}^{3}-3\;\mathrm{dph})$, $x_{ijklm4}(\mathrm{dph})=1/8(35\;{\mathrm{dph}}^{4}-30\;{\mathrm{dph}}^{2}+3)$, and $x_{ijklm5}(\mathrm{dph})=$ $1/8(63\;{\mathrm{dph}}^{5}-70\;{\mathrm{dph}}^{3}+15\;\mathrm{dph})$, ${\mathrm{animal}}_{l}$ is the random additive genetic effect of animal l (l=1 to 2156), and $e_{ijklm}$ is the random error term.

The model includes the fixed effects of strain, contemporary housing group, and gonadal sex. The filleting person was included in the model only for traits that were affected. The age curve was modeled by applying Legendre polynomials of degree 5. Polynomials of this order were chosen since we experienced convergence issues in some traits with lower-order polynomials. Polynomials of degree 5 yielded consistent results across all traits. We utilized the restricted maximum likelihood method as implemented in the WOMBAT software package (Meyer, 2007) to calculate variance components. The random effect of “animal” is distributed proportionally to the “arbitrary” relationship matrix, which has been supplied to WOMBAT as the inverse. Molecular relationship information was supplied to WOMBAT as a positive-definite, general inverse matrix file. Fixed effects were tested for significance for each trait by stepwise forwards selection comparing Akaikes information criterion and Bayes information criterion (AIC and BIC) values and by chi-square statistics (significance level: p=0.05) in the model adding one trait at a time. Estimates of correlations were obtained using the same model in a bivariate setting, since we experienced convergence issues with multivariate settings. Heritability estimates (ratio of additive genetic variance to phenotypic variance $h^{2}$) and genetic correlations including standard errors could be directly retrieved from WOMBAT output files.

## 3 Results

### 3.1 Descriptive statistics

Mean weights and ages for all strains at the start of the experiment are given in Table 1: fish of strain A (N=261) were the oldest and thus had the highest mean starting weight of 108.1 g. Although strains B (N=250) and D (N=862) were of comparable ages, Icelandic fish had a higher mean weight of 57.1 g. Starting weights of strains A and B, which originated from the first trial, had markedly higher standard deviations than fish in the second trial. While fish of strain D were around 70 d older than fish of strain C, both strains had similar mean weights.

**Table 2:** Descriptive statistics of all growth and carcass traits at slaughter for the complete dataset and separately for strains “Norway” and “France”. BW: body weight at slaughter (g); L: body length (cm); W: body width (cm); A: body area (cm${}^{2}$); FW: fillet weight (g), FY%: fillet yield (%), HE: head weight (g), LI: liver weight (g); GU: gut weight (g), HSI: hepatosomatic index; FCI${}_{\mathrm{PLN}}$: adapted Fulton's condition index; DP%: dressing percentage (%); GW: gutted weight (g); IW: weight of intestines (g); CA: weight of carcass discard (g); ADG: average daily gain between the ages of 300 and 500 dph; FCI: Fulton's condition index; Min: minimum, SD: standard deviation; Max: maximum; CV: coefficient of variation.

		BW	L	W	A	FW	FY%	HE	LI	GU	HSI	FCI${}_{\mathrm{PLN}}$	DP%	GW	IW	CA	ADG	FCI
Complete	Min	151.2	15.3	9.9	118.7	46.3	14.1	7.3	1.6	5.2	0.4	4.9	86.3	142.7	8.3	62.2	0.1	3.4
dataset	Mean	765.5	24.3	18.4	328.9	227.5	29.7	142.4	15.1	21.1	1.9	8.0	95.2	729.3	36.2	359.3	1.9	5.2
	SD	281.9	2.7	2.5	75.4	89.7	3.0	48.9	8.7	6.6	0.6	1.3	0.7	269.2	14.3	139.5	0.8	0.6
	Max	1910.0	33.0	27.1	661.8	616.5	38.0	327.0	77.2	50.8	10.2	19.2	97.6	1833.0	115.2	1948.0	4.7	8.8
	CV	36.8	11.1	13.6	22.9	39.4	10.1	34.3	57.3	31.7	33.4	17.3	0.8	36.9	39.4	38.8	40.8	12.3
Strain	Min	151.2	15.3	11.0	118.7	46.3	17.6	7.3	1.8	5.2	0.4	4.9	86.3	142.7	8.3	62.2	0.1	3.4
Norway	Mean	799.9	24.7	18.6	337.4	237.7	29.5	151.1	17.0	22.2	2.0	8.1	95.1	760.6	39.2	371.8	1.8	5.2
	SD	299.1	2.8	2.7	82.9	97.8	3.2	50.9	10.1	7.2	0.8	1.4	0.9	284.9	15.9	146.8	0.8	0.6
	Max	1910.0	33.1	26.7	661.8	616.5	38.0	327.0	77.2	50.8	10.2	19.2	97.6	1833.0	115.2	911.0	4.7	7.3
	CV	37.4	11.5	14.5	24.5	41.1	10.8	33.7	59.9	32.5	37.5	16.9	0.9	37.4	40.8	39.5	43.6	11.2
Strain	Min	186.6	16.1	9.9	137.9	57.9	14.1	39.8	1.6	6.4	0.4	5.1	93.2	177.5	9.1	79.8	0.2	3.6
France	Mean	787.6	24.5	18.5	329.1	232.5	29.5	146.1	13.4	20.8	1.6	8.3	95.6	753.3	34.2	374.7	2.2	5.4
	SD	235.2	2.1	2.0	58.3	72.9	2.8	39.9	5.5	5.4	0.3	1.3	0.5	225.7	10.3	119.9	0.7	0.6
	Max	1490.0	30.2	26.2	541.5	463.1	37.6	301.1	38.2	38.4	3.6	18.8	97.1	1432.0	70.6	741.9	4.4	7.9
	CV	29.8	8.6	11.0	17.7	31.3	9.6	27.3	40.9	25.9	20.5	16.1	0.5	29.9	30.1	32.0	32.2	11.6

Table 2 shows means, standard deviations, range, and the coefficient of variation (CV) for all traits at the time of slaughter. In the complete dataset, mean BW was 765.5 g with a high standard deviation of 281.9 g. Mean FW was 227.5 g, which translates to a mean fillet yield (FY%) of 29.7 %. The adapted Fulton's condition index (FCI${}_{\mathrm{PLN}}$) had an average of 8.0, which was higher than the conventional FCI with 5.2. ADG between the ages of 300 and 500 dph was 1.9 g. All weight-based traits showed high variation (CV: 31.7–57.3), whereas coefficients of variation for morphometric traits and calculated ratios were remarkably lower (CV: 0.8–22.9). For strain-wise comparison BW, FW, FY%, and morphometric traits were in similar ranges. Differences in viscera traits and ratios were mainly based on a higher mean liver weight (+3.6 g) in Norwegian fish. Standard deviations, and accordingly CVs, were generally higher for all traits in Norwegian fish due to higher age ranges within this strain.

### 3.2 Heritability estimates of growth and carcass traits

Heritability estimates in this study were low to moderate, ranging from 0.04 to 0.29 in univariate runs (Table 3). Heritability estimates for the economically most important traits BW, FW, and FY% were low with 0.19, 0.18, and 0.15, respectively. Estimates of heritability for individual carcass elements GU, CA, IW, and HE were also low between 0.14 and 0.19, with the exception of LI, which had a moderate heritability of 0.24. For morphological production traits heritability estimates were within the same range (L: 0.18 and A: 0.12), apart from W with a heritability of 0.08. A comparison of FCI (0.25) and FCI${}_{\mathrm{PLN}}$ (0.04) reveals a lower estimate for the latter condition index. ADG between the ages of 300 and 500 dph showed the highest heritability (0.29) of all traits we examined.

**Table 3:** Estimates of heritability ($h^{2}$) in the complete and strain-wise datasets: fixed effects are included in the model based on significance (chi-square: p<0.05). CG: contemporary group; date: date of slaughter; FP: filleting person, BW: body weight at slaughter (g); L: body length (cm); W: body width (cm); A: body area (cm${}^{2}$); FW: fillet weight (g), FY%: fillet yield (%), HE: head weight (g), LI: liver weight (g); GU: gut weight (g), HSI: hepatosomatic index; FCI${}_{\mathrm{PLN}}$: adapted Fulton's condition index; DP%: dressing percentage (%); GW: gutted weight (g); IW: weight of intestines (g); CA: weight of carcass discard (g); ADG: average daily gain between the ages of 300 and 500 dph; FCI: Fulton's condition index; standard errors are given in brackets, NC: no convergence.

Trait	Best-fit model includes age	Estimates of $h^{2}$	Estimates of $h^{2}$	Estimates of $h^{2}$
	(dph) as a covariable and	complete dataset	strain Norway	strain France
	the fixed effect of the following			
BW: body weight (g)	CG, date, origin, sex	0.19 (0.04)	0.27 (0.09)	0.31 (0.09)
L: body length (cm)	CG, date, origin	0.18 (0.04)	0.35 (0.10)	0.24 (0.08)
W: body width (cm)	CG, date, origin	0.08 (0.03)	0.16 (0.07)	0.12 (0.06)
A: body area (cm${}^{2}$)	CG, sex, origin	0.12 (0.03)	0.27 (0.09)	0.17 (0.06)
FW: fillet weight (g)	CG, date, FP, origin	0.18 (0.04)	0.25 (0.08)	0.29 (0.09)
FY: fillet yield (%)	CG, date, FP, origin, sex	0.15 (0.03)	0.14 (0.06)	0.23 (0.08)
HE: head weight (g)	CG, date, FP, origin	0.16 (0.04)	NC	NC
LI: liver weight (g)	CG, date, FP	0.24 (0.05)	NC	NC
GU: gut weight (g)	CG, date, FP, sex	0.14 (0.04)	NC	NC
HSI: hepatosomatic index	CG, date, sex, origin	0.18 (0.03)	NC	NC
FCI${}_{\mathrm{PLNadapted}}$ Fulton's condition index	CG, origin	0.04 (0.03)	0.00 (0.01)	0.12 (0.06)
DP: dressing percentage(%)	CG, date, origin	0.17 (0.04)	0.12 (0.05)	0.43 (0.10)
GW: gutted weight (g)	CG, date, FP, origin, sex	0.19 (0.05)	0.30 (0.09)	0.29 (0.08)
IW: weight of intestines (g)	CG, date, FP	0.20 (0.05)	NC	NC
CA: weight of carcass discard (g)	CG, date, FP, origin, sex	0.19 (0.04)	NC	NC
ADG: average daily gain	CG, origin	0.29 (0.05)	0.16 (0.07)	0.36 (0.10)
FCI: Fulton's condition index	CG, origin	0.24 (0.05)	0.29 (0.09)	0.27 (0.09)

Separate analysis of strains using the same models generally resulted in higher heritability estimates, particularly for BW (0.27–0.31), FW (0.25–0.29), and FY% (0.14–0.23). Standard errors of all estimates were slightly higher than in the complete dataset. Estimates for biometrical traits were also higher in both separate strains, although trends and patterns between those traits remained the same with W (0.12–0.16) showing the lowest estimates and L (0.24–0.35), ADG (0.16–0.36), and DP% (0.12–0.43) showing the highest heritabilities. While estimates for FCI were similar between strains, heritability for FCI${}_{\mathrm{PLN}}$ was virtually zero in Norwegian fish and 0.12 in French fish, indicating that low and unreliable estimates for this trait in the complete dataset are based on one strain.

**Table 4:** Estimates of phenotypic ($r_{p}$) and genetic ($r_{g}$) correlations for body weight at slaughter (BW) and other traits. BW: body weight at slaughter (g); L: body length (cm); W: body width (cm); A: body area (cm${}^{2}$); FW: fillet weight (g), FY%: fillet yield (%), HE: head weight (g), LI: liver weight (g); GU: gut weight (g), HSI: hepatosomatic index; FCI${}_{\mathrm{PLN}}$: adapted Fulton's condition index; DP%: dressing percentage (%); GW: gutted weight (g); IW: weight of intestines (g); CA: weight of carcass discard (g); ADG: average daily gain between the ages of 300 and 500 dph; FCI: Fulton's condition index; standard errors are given in brackets; NC: no convergence.

Body weight	Phenotypic	Genetic
(g) and the following	correlations: $r_{p}$	correlations: $r_{g}$
Fillet weight (g)	0.95 (< 0.01)	0.95 (0.02)
Head weight (g)	0.90 (< 0.01)	0.97 (0.02)
Liver weight (g)	0.79 (< 0.01)	0.79 (0.06)
Gut weight (g)	0.84 (< 0.01)	0.76 (0.07)
Weight of carcass discard (g)	0.98 (< 0.01)	0.99 (< 0.01)
Body length (cm)	0.89 (< 0.01)	0.89 (0.04)
Body width (cm)	0.70 (0.01)	0.99 (0.05)
Body area (cm${}^{2}$)	0.86 (< 0.01)	0.96 (0.03)
Fulton's condition index	0.38 (0.02)	0.48 (0.13)
Adapted Fulton's condition index	0.21 (0.02)	0.75 (0.17)
Gutted weight (g)	NC	NC
Average daily gain	0.79 (< 0.01)	0.88 (0.03)
Fillet yield (%)	0.03 (0.02)	0.11 (0.17)
Hepatosomatic index (%)	0.25 (0.02)	0.41 (0.14)
Weight of intestines (g)	0.89 (< 0.01)	0.85 (0.04)
Dressing percentage (%)	0.05 (0.02)	0.15 (0.16)

### 3.3 Genetic and phenotypic correlations between traits

Genetic and phenotypic correlations between BW (the target trait for selection) and all other traits are given in Table 4, while a complete table of all correlations for all traits can be found in the Supplement (Table S2). Phenotypic correlations between BW and other weight-based, as well as morphometric, traits were generally high (0.70–0.94). FW, HE, and CA had phenotypic correlation estimates higher than 0.9. Genetic correlations for these combinations were also above 0.9. Of the morphometric traits A and W had the highest positive correlations (> 0.9) with BW. While phenotypic correlations of both condition indices with BW were weak, we found moderate positive genetic correlation for BW–FCI${}_{\mathrm{PLN}}$. Genetic correlations between FCI${}_{\mathrm{PLN}}$ and other traits were always higher than the ones between FCI and other traits. Genetic correlation between BW and FW was also high (0.95), while it was very low with large standard errors for fillet yield (0.11±0.17) and dressing percentage (0.15±0.15). Ratio traits expressed in percentages and both condition index traits often produced estimates difficult to interpret for both genetic and phenotypic correlations, since standard errors were often higher than the estimate itself. Additionally, negative estimates solely occurred for correlations involving those traits. The three biometric traits L, W, and A were highly genetically correlated with FW ($r_{g}$: 0.86–0.94) and GW ($r_{g}$: 0.89–0.99). Both genetic and phenotypic correlations for BW–AVG were high ($r_{p}$: 0.79 and $r_{g}$: 0.87). Due to model convergence problems correlations between traits BW–GW, FW–AVG, HE–CA, HE–W, and CA–AVG could not be estimated.

### 3.4 Discussion

The heritability estimates we obtained in this study are low to moderate, indicating potential for improvement via selective breeding programs. A number of studies on genetic parameters for body weight at harvest exist in turbot, which generally report slightly higher estimates. Sánchez-Molano et al. (2011) reported heritability estimates of 0.45 for body weight in their study. Xu et al. (2015) estimated heritability estimates ranging from 0.2 to 0.4 for body weight under different temperature regimes, whereas Gjerde (1997) reported higher estimates ranging from 0.4 to 0.7. We suspect that our results might be slightly underestimated due to both the use of molecular relatedness and the joint analysis of multiple strains. So far, there are only two publications using MR to estimate genetic parameters in flatfish: Blonk et al. (2010) calculated heritability for body weight in common sole (0.11–0.13), while Guan et al. (2015) compared the MR method to the classical pedigree-based approach and found heritability estimates for body weight at 100 dph of 0.19 (MR) and 0.66 (pedigree). Lower values for estimated variance components based on molecular relatedness have also been reported in a number of other species (e.g., Thomas et al., 2002; van Kleunen, 2005) and have been potentially linked to the denseness of the relationship matrix. Powell et al. (2008), among others, discussed a lack of comparability of different populations within the same species, which might partly be due to genetic differences in founder animals. Although the calculation of the genomic relationship matrix includes a standardization step to minimize founder effects, we received higher estimates in all traits when we analyzed strains separately, an effect that can be expected for within-family variance estimation. Since most turbot breeding programs in the different European countries treat their populations as separate from each other, these results could be considered more realistic.

To our knowledge, this is the first time that genetic parameters for carcass and fillet traits are being reported for this species. Despite the obvious difficulties in direct comparability with other aquaculture species, particularly roundfish, it can be stated that our results seem to be within a reasonable range. Saillant et al. (2009) reported heritability estimates of 0.25 for FY% in sea bass, while Navarro et al. (2009) presented heritability of 0.15 for FW and 0.12 for FY% in gilt-head sea bream. Neira et al. (2004) found very similar estimates (FW: 0.18, FY%: 0.11) in coho salmon, whereas heritability estimates in other salmonids are usually much higher (e.g., Powell et al., 2008; Atlantic salmon FW: 0.52). Many of the above studies reported problems in the analysis of mathematically derived ratio traits, which we experienced as well, especially regarding correlations involving those traits. Powell et al. (2008) and Rutten et al. (2005), in particular, argued the usefulness of yield traits as selection criteria since ratios are calculated from closely proportional traits. Consequently, we tried an alternative, indirect method to calculate heritability for fillet yield by including body weight as an extra covariable in the model for fillet weight, but we received close to identical results from both methods (results not shown here). Alternative methods to measure and quantify ratio traits are needed to make them more useful as selection target traits. Efforts have been made in other livestock species to develop direct, in vivo methods for fillet or carcass yield determination based on X-rays, computer tomography, or ultrasound (e.g., Sather et al., 1996; Milisits et al., 1999).

Genetic correlations between weight-based traits that were directly measured were all positive and high (> 0.7) (for example, BW at harvest, GW, or FW). We also found high positive correlations for the target traits BW and FW with morphometric traits. The highest correlations were found for A, which makes it a very suitable alternative selection trait. The use of morphometric traits such as length, width, or area as alternative selection criteria is particularly interesting since selection candidates do not have to be sacrificed to determine these measurements. With the advance of noninvasive, camera-based measurement techniques handling stress could be further minimized as well.

Another interesting selection trait could be ADG, since it is comparatively highly heritable (0.3) and strongly genetically correlated with body weight at harvest (0.88), but it can be measured nonlethally at significantly earlier life stages (300–500 dph in this case).

While a prior study (Arfsten et al., 2010) suggested the adapted condition index for Pleuronectiformes FCI${}_{\mathrm{PLN}}$ as a useful selection trait based on high phenotypic correlations with BW, we could not confirm this finding. When compared to the classical Fulton's condition index, the adapted FCI${}_{\mathrm{PLN}}$ showed higher genetic correlations with other traits. This resulted in lower $h^{2}$ estimates since a large part of the variance is already explained by the variance of the underlying traits. Like with other types of yield and ratio traits this makes them less suitable for direct selection.

## 4 Conclusions

This study presents heritability estimates and genetic correlations for weight and carcass traits in turbot. Estimates of heritability for weight-related traits were moderate, thus indicating that there is inter-individual variation and selection on these traits is beneficial. We were able to demonstrate the usefulness of biometrical body traits as alternatives for selection and found no potential for inadvertent negative selection response between body weight and carcass quality traits.

## Supplement

10.5194/aab-62-265-2019-supplement The supplement related to this article is available online at: https://doi.org/10.5194/aab-62-265-2019-supplement.

## Data Availability

Access to genetic and phenotypic data used in this study will be provided upon request to Jens Tetens (jens.tetens@uni-goettingen.de).
