# Supplementary material for: Estimation of genetic parameters for growth and carcass traits in turbot (Scophthalmus maximus)
Source: Arch Anim Breed. 2019 May 6;62(1):265–73. doi: 10.5194/aab-62-265-2019 (PMC6852839; doi:10.5194/aab-62-265-2019)
Supplement: The supplement related to this article is available online at: https://doi.org/10.5194/aab-62-265-2019-supplement. [file aab-62-265-supplement.pdf]

Supplement of Arch. Anim. Breed., 62, 265–273, 2019
https://doi.org/10.5194/aab-62-265-2019-supplement
© Author(s) 2019. This work is distributed under
the Creative Commons Attribution 4.0 License.

Open Access
Archives Animal Breeding

Supplement of

Estimation of genetic parameters for growth and carcass traits in turbot (*Scophthalmus maximus*)

Kristina Schlicht et al.

Correspondence to: Kristina Schlicht (kristina.schlicht@uksh.de)

The copyright of individual parts of the supplement might differ from the CC BY 4.0 License.

Table S1: Information on markers and primers used for genotyping. Marker: marker name; Acc. No: accession number; FrL: mean fragment length (Bp); Direction: Forward (F) or reverse (R); Primer Sequence; T.opt: optimal annealing temperature; Location on linkage group (LG) in centiMorgan (cM), PIC: polymorphism information content in test subjects; No of alleles observed.

Marker	Acc. No	FrgL	Direction	Primer Sequence	T.opt	LG	cM	PIC	No allleles
**Sma-E158**	EY455452.1	112	F	AAAGCAGATGTTGCAGACGCAG	57.67	16	9	0.428	3
			R	CGAGAATTTTTACGAGCGACGGCA	59.93				
**Sma-USC28**	dq470765	320	F	GAGGCCCGCAGAGACAGAGGTA	60.13	6	28.8	0.881	11
			R	GCGGTATGAACCCCGGCCATTG	60.21				
**Sma-USC147**	dq810947	218	F	AGGACACAAGCACTGGGTCACA	60	6	19.5	0.586	5
			R	ACGCCTGGGTGAGTCACGTCAA	59.96				
**SmaUSC-E12**	FE947465	161	F	TTGACCAGTGCCAAGCACAGCG	59.96	17	67.1	0.559	4
			R	ACCTGTTTCCACTTCTCTCCCTCGT	59.87				
**Sma-USC63**	DQ810863	279	F	CACCCTGGTGTGGCATCACGC	60.1	14	32.1	0.77	7
			R	CCGTTCTGCCCCCGACTAGACA	59.95				
**Sma-USC175**	DQ810975	370	F	TCTCGCCAGACAATGTCACCGT	59.99	10	0	0.636	5
			R	TCGCTCACCTGGACATCGGGAG	60				
**Sma-USC217**	DQ811017	199	F	CGCCCCATCTCCCCAACCTCTT	60.37	10	10.1	0.705	7
			R	CCACGGCGAGTCATTGTGGTGG	60.13				
**Sma-USC249**	dq811049	119	F	AGAGAAAGAGAAGAGACACCCACC	59.84	2	44.8	0.726	5
			R	AGGCCTGTCGCTGTTTGCATTG	60.01				
**Sma-E164**	EY456203.1	300	F	TTTTGGCCCTTTTCCGCTGGCA	60.04	14	60.7	0.52	4
			R	ACCGGTAGCCTTTTGTGATTTCCA	60.25				
**Sma-E117**	HS031010	143	F	TGCGAGTGTGCGTGTGTTGGAT	59.7	9	58.6	0.337	2
			R	GCCGCATTTGAAATATCATCGTGGC A	59.28				
**Sma-USC273**	dq811073	215	F	TTGGGTCCCTTGTCAAGCAGCA	60.14	23	11.2	0.496	3

			R	TGACAGCCCTGCTCGCACTGTA	59.7				
**Sma-USC91**	DQ810891	268	F	GACCATGCGCCACTTCTTGGCT	59.47	17	5.4	0.803	7
			R	GGCAGAGGGAGAGGAGAGCGTT	59.72				
**Sma-USC136**	DQ810936	118	F	AGCCTCATATAGCGGTGCCATGC	59.18	16	41	0.555	3
			R	TTGATCCATCTGTGCGCTCCGC	58.73				
**SmaUSC-E19**	FE944607	184	F	TCACGTTGCCCCTCAGCTCCTT	59.84	18	25.9	0.51	4
			R	TGTACTGGTGACTCGGAGGGGG	59.85				
**SmaUSC-E38**	FE948185	311	F	TCTGCTCAGCCAACCTCACCGT	60.25	13	58.9	0.712	6
			R	ACAGTCCCATGCAGAAATAACCGT	60.1				
**Sma-USC-E33**	fe947436	185	F	TGCCTTGGTCTGAGGGGAGTCA	60.07	23	46.9	0.629	5
			R	TGACGATGCTGCTGACACGACG	60.05				
**Sma-USC165**	DQ810965	92	F	CGGGCTGGGTAAAGCATGCACA	59.76	11	40.3	0.553	6
			R	GCCGGCGATCCCGTAAACAGAA	60.06				
**SmaUSC-E43**	FE945769	103	F	TTTACGGCCCTCCTCTGCGTGT	60.21	8	14.3	0.525	5
			R	GGGTTTCATTCCCGCTCTGGGC	60.03				
**Sma-USC229**	dq811029	223	F	TTGTCATTCACGTGCGGAGGCG	59.96	24	6.7	0.853	9
			R	CCGCTGCGGCTTCATCTTTTCT	59.86				
**SmaUSC-E1**	FE944126	151	F	AGGAGGAACGGAGCAGGACACG	60.74	17	55.8	0.72	6
			R	GGAGCCTCGTCACCGGTCTGAA	60.5				
**Sma-USc210**	dq811010	224	F	ACGTGTCAAACTTCTCTTTGTTCTCG T	60	24	0	0.691	6
			R	GCAGCCACCAACAGCATCGCAA	60.05				
**Sma-USC253**	DQ811053	79	F	ATAATGACGCCACGGGCCAAAG	60.26	14	55.7	0.51	4
			R	GGGGTTGGACGGGTTCCTCCTT	59.57				
**Sma-USC157**	dq810957	96	F	TTCTCCCCTCTTGCTGTATGCG	59.97	3	38	0.612	4
			R	TGGACTGGAGCTGAAGCAAGCA	60.86				
**Sma-USC50**	DQ810850	317	F	AGTCCAAGTGTGAACGTACTGCCT	59.65	16	55.6	0.78	8
			R	ACAAAAGCCCGAGGCAGCGTTT	60.17				
**Sma USC-E42**	FE945758	103	F	CCGAGCTGTTCGAAGCCTCCAT	59.97	1	0	0.73	7
			R	CAACACCGCACAGACCTGCGAT	58.62				

**Sma-USC176**	DQ810976	196	F	TCGCGTGAAAGCAAAATTGGATGC	59.72	20	0	0.313	4
			R	TGTGCCACATACTCGGCAAACTGA	59.5				
**Sma-USC269**	DQ811069	256	F	AGTGGAGGATGTTATAAATGGACG GGT	60.11	8	29.4	0.723	6
			R	GCAGTGTCCCCTGAAGCCATGT	58.6				
**Sma-USC201**	dq811001	202	F	ACCTCGAGACCTCGCTTACCTCA	60.65	11	7	0.551	3
			R	GGGCCCTGTGCCGTATCACAAC	60.4				
**Sma-USC108**	DQ810908	298	F	CATCGTGCCTCCATACACGCCC	60.33	19	10.6	0.501	3
			R	AGCACCGGAGTCAGCATAGGCA	59.97				
**Sma-USC65**	dq810865	120	F	AGGTGCAGAGGAGCGACATGGT	59.98	5	18.1	0.359	2
			R	TCGTGATCTACAGTGCGCCGCT	60.13				
**Sma-USC194**	DQ810994	116	F	TGAGACCATCACAACTGCCCGT	59.87	8	3.7	0.845	10
			R	TGTGTAATCGGAATGCGCTCGG	60.01				
**Sma-E183**	HS029459	194	F	GACATGAAACAGGAAGGGAACAGC A	58.4	16	57.2	0.818	8
			R	ACACCTCAGGATAGCAATTATTGGC AC	59.87				
**smax03**	z78098	250	F	ATGCCTCTGGGGGACACAGACA	60.07	3	70	0.595	5
			R	TCCTTTTTGTTTGCCCATGACAACCC	59.82				
**Sma-E167**	EY977553.1	268	F	CGACATGACGGCACTGGGCATC	59.98	22	0	0.826	9
			R	CTGTTTTAATTACGTTTGTGAGTCGT CTGG	59.95				
**Sma-USC228**	dq811028	119	F	AGGCTCATCGACTGCACTGTCA	60.2	1	30.7	0.635	4
			R	AGAGCGTTCGCTTTTGTCCGGC	59.81				
**Sma-USC284**	DQ811084	218	F	GGTGCAAAGTGGAAGTTGCAAAGT TTTCT	58.84	20	35.6	0.728	6
			R	ACAGGATGGACAATGACTGACCCA A	60.07				
**Sma-E283**	hs030649	110	F	TGTGAATTTAGGAGCACAGGCAAC A	59.58	4	10	0.853	9
			R	ACCTGGTATCTTCACCCCTGTCCCT	59.94				

**Sma-USC172**	DQ810972	150	F	TCACCCTGCGAGAAGGAGAGGG	59.65	16	20.6	0.636	5
			R	CTTTGATGGGTGACTCCAAGAATCGC	59.88				
**Sma-USC143**	DQ810943	296	F	AGCCGTTTTGTAGTGTTTGAGTGAGC	59.5	12	30.1	0.744	7
			R	GGGCTGTTTTCCATTATTCGGGCA	59.97				
**Sma-USC235**	DQ811035	176	F	ACCCGTCTAAAAGAGACGTTGGTGT	60.26	11	50.7	0.553	6
			R	GTCGGCATGTGGACTGGTGGAG	60.04				
**Sma-USC56**	DQ810856	111	F	GCCAGGGACCTTGTTCCAAACCC	60.06	12	26.8	0.443	4
			R	GTCAACCAGGGCTAGATCTCTCCACA	59.5				
**Sma-USC98**	dq810898	266	F	GCCAGTCAATCTCAGCCAACGGG	60.21	3	76.5	0.776	8
			R	GGCTGGCTCACAACGGCAACAT	59.94				
**Sma-USC92**	dq810892	267	F	GAAGGCGGGGGAAGGTCTAACA	59.14	20	30.6	0.555	3
			R	TGCCTGCATATTGACGCGAGGG	60.12				
**Sma-USC45**	DQ810845	191	F	AGCAGGACAGCGAGACAGTCGT	59.95	15	16.7	0.605	4
			R	CCTTGCTTCCCTGCGCCTGATT	59.89				
**SmaUSC-E23**	FE945024	112	F	TGGCACAAATCCCCCTCCCGAA	59.87	9	29.3	0.855	11
			R	GGAGTTGCACAAAGGGAAGATTCTCCA	59.99				
**Sma-USC258**	DQ811058	120	F	GCCTCCTGTGAGAGAATCAGAGCCA	59.78	11	4.3	0.735	7
			R	ACTGGGCAAAGCACACGGAAGG	60.25				
**SmaUSC-E27**	FE951828	179	F	ACTAGCTGGTGTAGCCGCTGTGA	60.16	10	26.5	0.68	6
			R	TCTTGCAGCAGAACTGTGTCCACT	59.91				
**Sma-USC117**	DQ810917	272	F	AGGGCTTTGTGAGCTCTGGTCG	59.8	21	13.3	0.816	7
			R	CTGGCCATCGTCCCGTCTTTGT	59.98				
**Sma-USC230**	dq811030	200	F	TCTCCCAGTCTCTCTCTCTCCCTCT	60.01	4	63.7	0.658	4
			R	CTTCAGCATGCCTTACATGTGTGC	59.57				
**Sma-E284**	HS031426	275	F	GGGCAGAAGTTAAGTGTGTTGCCT	60.52	11	24.4	0.447	3
			R	AATCTGGCAGTCCAAGCAGCCC	60.81				

**Sma-USC161**	dq810961	135	F	GCATCATGCCGACAGCTGCACTA	59.67	2	22.9	0.748	6
			R	GCACTCGATGAACCCGCCCTTT	60.04				
**Sma-USC162**	DQ810962	164	F	GGCACCTTGCAACAGCTGGTCT	59.54	10	35.1	0.51	4
			R	TCCCTGACCTGTTGCTCCCCTC	59.93				
**Sma-USC250**	DQ811050	195	F	TGCGCACATTGACTGGAAGCCT	60.97	16	31.8	0.714	7
			R	GCCAGTGTAAGCATCGGCAGGG	60.14				
**SmaUSC-E4**	FE949040	103	F	GGCCCAGCTCTGTGTCTCCACT	60.02	8	47.8	0.667	4
			R	GCCGAAACAACCTCTGCGGCAT	59.82				
**Sma-USC247**	dq811047	278	F	AAGGGGTTTCGGGGAGGGCG	59.96	5	36.1	0.68	6
			R	TTCCAGACCGGAGGCTGCTGAA	60.32				
**Sma-USC52**	DQ810852	225	F	CTGGGATCTGCTCCCCCGGTTT	58.91	17	27.8	0.735	7
			R	CGACTACACAGCGACCCAGTGC	60.06				
**Sma-USC222**	dq811022	161	F	CGGCTGTAACTTCCTGTCGCCC	60.02	1	91.5	0.398	3
			R	TCATCAGCGCAGCAGCAGCAAA	61.08				
**Sma-USC93**	dq810893	253	F	TACCTGACGGCACTCGACAGCA	59.87	3	0	0.653	5
			R	GTGATCGCATCACACTTTCCCTGT	59.2				
**Sma-USC214**	DQ811014	180	F	CTCCGTGTGCGGTGGGTTCATC	60.34	15	8.8	0.807	8
			R	AACCTGGGGGTCAGGACAAGGG	60.32				
**Sma-USC220**	DQ811020	112	F	TGATCCCAGTTTGACTGAGGCAGC	59.59	14	8.8	0.668	5
			R	CACTGACCTTCAGCGTGCGTGT	60.91				
**Sma-USC266**	DQ811066	239	F	CAGATCCGGCCTGCTCAGCACA	59.87	12	60.3	0.636	5
			R	TCCCGCCCAGCGTGTTCAGATG	59.14				
**Sma-USC226**	DQ811026	174	F	GGTTCCAGTGGATGCTGAGTTGTGT	59.81	9	70.3	0.612	6
			R	GAGAGGGAGCAGCTGGGGGTTT	59.56				
**Sma-USC7**	aj224993	292	F	CAGATATCCACCGGCGGAGGGG	60.89	4	48.8	0.712	6
			R	GTGGGGCACGCGGTGTCATATT	59.89				
**Sma-E218**	HS032074	77	F	ATAACTCACCCGCACTGGACCG	59.91	8	10.1	0.584	5
			R	GGCTGAGGCTGGACACCAAGAC	60.12				
**Sma-USC22**	DQ470759	248	F	TCCAGCTTGAACGCACCTTATCCT	59.82	11	43	0.835	9

			R	AGGATTTGTTTGGGGCACAGGGT	60.97				
**Sma-USC26**	DQ470763	181	F	CCAACGGACTAACAAACAGACCGGA	60.14	15	45.7	0.816	8
			R	GCTGATGTCCTTTCGGGGAGGC	60.02				
**Sma-USC271**	DQ811071	217	F	AGCTGGTGGAGTGGAAAGCCCA	59.82	1	56.4	0.68	6
			R	ACCAGCGTAACTCGTGTGCTGA	59.96				
**Sma-USC11**	DQ470748	175	F	ACACAGACAGAGATAGAGGGTGAGGGT	60.32	10	41.2	0.787	7
			R	TCCTGCCTTCACAGACGCCCAT	58.91				
**Sma-USC96**	DQ810896	281	F	TCGAGCGTGGGCAAATGTCAGG	59.82	10	61.2	0.641	5
			R	GCAAGTTGCTTGCTGAATATGGCA	59.98				
**Sma-USC280**	DQ811080	117	F	ACAAAAGCGCTCTCTGTCGCCC	59.95	13	0	0.835	9
			R	CCTTTATGCAGCTCTTTCATCCCCC	60.2				
**Sma-USC13**	DQ470750	185	F	TGCAGGGCCATTTGTGGCACTTT	59.81	1	86.9	0.748	6
			R	AGAGGCAGAGCAACATTATGGGGA	58.84				
**Smax-03**	Z78098	250	F	ATGCCTCTGGGGGACACAGACA	60.07	3	70	0.617	5
			R	TCCTTTTTGTTTGCCCATGACAACCC	59.58				
**4/4AC4/13**	AF182094	235	F	CCGGCTGTGAGCTGTCATGATGT,	59.94	12	8.1	0.755	5
			R	AGAGGGACCGTGAGGGAGACAGA	59.65				
**Sma-USC27**	DQ470764	272	F	GCGTTAGCGCATTACCGCCATC,	60.04	13	0	0.871	10
			R	GCGGAAGAAGATACTGTCTCACTACA	60.25				
**Smax-02**	Z78101	111	F	TGGGAGATGGAGGCAATGGAGGA,	59.7	17	12.8	0.759	7
			R	CAGAGCAGGTCATTATACAGCTAGAGG	59.28				
**Sma-USC48**	DQ810848	149	F	TCAGCCAACACCTTTGGTGCCC,	60.14	8	42.1	0.772	7
			R	TGCATTGACAAAAGACGCGGGGA	59.7				
**Sma-USC213**	DQ811013	296	F	ACATGTCCGTCCACGTGTCCCT,	59.47	14	38.4	0.854	10
			R	AATGCCTACACCCGGACGGTCT	60.07				
**2/5TG14**	AF182089	231	F	GGCCGCCCTGAACAACATGGAG,	60.05	19	20.7	0.748	9

			R	AAAGGACCGGAGCATCCCTCCC	59.76				
**Sma-USC15**	DQ470752	294	F	CCAACAGCCTCCAGCCAATCCC,	60.06	1	44.8	0.766	6
			R	TCCCCCAGGGAGAGCAGAGAGA	59.97				
**Sma-USC29**	DQ470766	128	F	CCCCACAAAAACACTGAATCCCAG G,	60.26	20	5.1	0.692	7
			R	AGCTGGACAAGGAAACAGCCGC	59.57				
**Sma-USC35**	DQ470772	162	F	TGCATCATGGAGCCAAAAGGCAC,	59.97	12	4.9	0.755	7
			R	GCTGGCACTGTGGGATGCAGAG	60.86				
**7/1TC18**	AF182097	214	F	GCGACCCACTGCAGTTACACTCT,	59.65	6	90.1	0.768	6
			R	TGCACTGCAGCAGACACACATCA	60.17				
**Sma-USC81**	DQ810881	213	F	CGCCCCAGAAGACAACAGGCAG,	60.13	14	26.4	0.89	12
			R	TGCGAAACTGAACTCAGGACACA	60.21				
**Sma-USC221**	DQ811021	123	F	TCCCTCCCGTGCAGACTAGGAGA,	60.1	15	52.1	0.759	10
			R	GTCGACGAAACTTCGCTGACCT	59.95				
**Sma-USC10**	DQ470747	147	F	CCCCACAAAAACACTGAATCCCAG G	59.99	5	61.4	0.679	5
			R	GATGGACCGGAGCTTAGCCAGC	60.07				

Table S2: phenotypic and genetic correlations for all traits: r_g are presented in lower matrix triangle, r_p are presented in upper matrix triangle; standard errors are given in brackets; trait abbreviations can be found with Table 2; NC: no convergence

	BW	FW	HE	LI	GU	CA	L	W	A	FCI	FCI_PLN	GW	AVG	FY%	HSI%	IW	DP%
**BW**		0.946 (0.002)	0.900 (0.004)	0.788 (0.008)	0.843 (0.006)	0.977 (0.001)	0.898 (0.004)	0.704 (0.011)	0.856 (0.006)	0.377 (0.019)	0.210 (0.021)	NC	0.793 (0.008)	0.034 (0.022)	0.245 (0.021)	0.892 (0.005)	0.046 (0.022)
**FW**	0.949 (0.017)		0.812 (0.008)	0.757 (0.010)	0.782 (0.009)	0.871 (0.005)	0.843 (0.006)	0.664 (0.013)	0.810 (0.008)	0.358 (0.019)	0.196 (0.022)	0.946 (0.002)	NC	0.331 (0.020)	0.236 (0.021)	0.842 (0.006)	0.050 (0.022)
**HE**	0.967 (0.018)	0.873 (0.047)		0.645 (0.013)	0.764 (0.009)	NC	0.845 (0.006)	NC	0.778 (0.009)	0.292 (0.020)	0.192 (0.022)	0.902 (0.004)	0.631 (0.013)	-0.081 (0.022	0.143 (0.022)	0.766 (0.009)	0.111 (0.022)
**LI**	0.787 (0.060)	0.745 (0.069)	0.713 (0.088)		0.663 (0.013)	0.762 (0.009)	0.647 (0.013)	0.530 (0.016)	0.633 (0.013)	0.400 (0.019)	0.209 (0.021)	0.776 (0.009)	0.470 (0.017)	0.035 (0.022)	0.726 (0.011)	0.926 (0.003)	-0.405 (0.019
**GU**	0.760 (0.071)	0.666 (0.096)	0.715 (0.089)	0.692 (0.093)		0.817 (0.007)	0.772 (0.009)	0.591 (0.015)	0.726 (0.011)	0.326 (0.020)	0.199 (0.022)	0.835 (0.007)	0.574 (0.015)	-0.022 (0.022	0.208 (0.021)	0.896 (0.004)	-0.252 (0.021
**CA**	0.987 (0.005)	0.892 (0.036)	NC	0.749 (0.065)	0.754 (0.074)		0.871 (0.005)	0.692 (0.012)	0.836 (0.007)	0.376 (0.019)	0.202 (0.022)	0.977 (0.001)	NC	-0.117 (0.022	0.230 (0.021)	0.863 (0.006)	0.059 (0.022)
**L**	0.887 (0.035)	0.861 (0.045)	0.910 (0.037)	0.647 (0.093)	0.657 (0.098)	0.584 (0.044)		0.805 (0.008	0.906 (0.004)	0.132 (0.021)	0.115 (0.021)	0.899 (0.004)	0.727 (0.010)	0.077 (0.021)	0.154 (0.022)	0.772 (0.009)	0.110 (0.022)
**W**	0.993 (0.045)	0.946 (0.081)	NC	0.707 (0.128)	0.667 (0.135)	0.928 (0.062)	0.913 (0.026		0.930 (0.003)	0.249 (0.020)	-0.314 (0.019)	0.704 (0.011)	0.646 (0.013)	0.019(0.022)	0.151 (0.022)	0.612 (0.014)	0.072 (0.022)
**A**	0.963 (0.025)	0.926 (0.039)	0.965 (0.034)	0.691 (0.097)	0.725 (0.094)	0.931 (0.033)	0.957 (0.011)	0.980 (0.009)		0.240 (0.020)	-0.107 (0.021)	0.857 (0.006)	0.732 (0.010)	0.031 (0.022)	0.155 (0.022)	0.741 (0.010)	0.097 (0.022)
**FCI**	0.478 (0.127)	0.406 (0.134)	0.341 (0.149)	0.468 (0.118)	0.420 (0.149)	0.535 (0.122)	0.328 (0.107)	0.397 (0.107)	0.357 (0.102)		0.428 (0.018)	0.373 (0.019)	0.397 (0.018)	-0.003 (0.022	0.258 (0.021)	0.401 (0.019)	-0.112 (0.022
**FCI_PLN**	0.751 (0.172)	0.604 (0.197)	0.677 (0.205)	0.761 (0.176)	0.719 (0.220)	0.835 (0.158)	0.381 (0.111)	0.152 (0.129)	0.245 (0.118)	1.000 (0.145)		0.208 (0.021)	0.207 (0.021)	-0.001 (0.022	0.124 (0.022)	0.224 (0.021)	-0.058 (0.022
**GW**	NC	0.951 (0.017)	0.967 (0.017)	0.771 (0.064)	0.747 (0.075)	0.986 (0.005)	0.889 (0.035)	0.991 (0.048)	0.964 (0.025)	0.473 (0.127)	0.744 (0.174)		NC	0.035 (0.022)	0.228 (0.021)	0.880 (0.005)	0.068 (0.022)
**AVG**	0.877 (0.026)	NC	0.703 (0.085)	0.609 (0.099)	0.601 (0.111)	NC	0.804 (0.040)	0.756 (0.053)	0.795 (0.040)	0.573 (0.078)	0.511 (0.090)	NC		-0.081 (0.022	0.119 (0.022)	0.567 (0.015)	0.050 (0.022)
**FY%**	0.111 (0.173)	0.411 (0.149)	-0.045 (0.179)	0.074 (0.162)	-0.113 (0.189)	-0.021 (0.174)	0.052 (0.127)	0.210 (0.219)	0.159 (0.189)	-0.073 (0.164)	-0.238 (0.269)	0.116 (0.172)	-0.139 (0.161)		0.009 (0.022)	0.010 (0.022)	0.047 (0.022)
**HSI%**	0.413 (0.143)	0.347 (0.147)	0.363 (0.161)	0.877 (0.046)	0.390 (0.166)	0.410 (0.145)	0.314 (0.156)	0.263 (0.204)	0.256 (0.173)	0.319 (0.139)	0.600 (0.221)	0.392 (0.145)	0.374 (0.144)	-0.030 (0.168)		0.535 (0.016)	-0.762 (0.009
**IW**	0.846 (0.043)	0.782 (0.060)	0.772 (0.071)	0.952 (0.017)	0.880 (0.040)	0.829 (0.049)	0.704 (0.080)	0.746 (0.111)	0.766 (0.077)	0.485 (0.124)	0.788 (0.175)	0.830 (0.048)	0.658 (0.093)	0.003 (0.172)	0.750 (0.090)		-0.367 (0.019
**DP%**	0.147 (0.159)	0.226 (0.159)	0.175 (0.164)	-0.391 (0.128)	-0.366 (0.162)	0.134 (0.160)	0.216 (0.156)	0.274 (0.197)	0.268 (0.169)	-0.112 (0.022)	-0.248 (0.248)	0.175 (0.158)	-0.038 (0.153)	0.289 (0.164)	-0.602 (0.093	-0.404 (0.136)	

Table S3: Index of abbreviations used in the text:

Abbreviation	Full Term
MR	Molecular relatedness
FCI	Fultons Condition Index
$FCI_{(PLN)}$	adapted Condition Index for Pleuronectiformes
ADG	Average Daily Gain
h^2	Heritability
HSI	Hepatosomatic index
REML	Restricted Maximum Likelihood
dph	Days post Hatch
BW	Body weight
L	Body length
W	Body width
A	Body surface area
RAS	Recirculating Aquaculture System
CG	Contemporary housing group
FP	Filleting Person
FW	Fillet weight
FY%	Fillet yield
HE	Head weight
LI	Liver weight
GU	Stomach and gut weight
CA	Carcass weight
IW	Weight of intestines
GW	Gutted weight

DP%	Dressing percentage
S	Similarity index
CV	Coefficient of variation
NC	No convergence
